# Mslar: Microbial synthetic lethal and rescue database

**DOI:** 10.1371/journal.pcbi.1011218

**Published:** 2023-06-08

**Authors:** Sen-Bin Zhu, Qian-Hu Jiang, Zhi-Guo Chen, Xiang Zhou, Yan-ting Jin, Zixin Deng, Feng-Biao Guo

**Affiliations:** 1 School of Life Science and Technology, Center for Informational Biology, University of Electronic Science and Technology of China, Chengdu, China; 2 Key Laboratory of Combinatorial Biosynthesis and Drug Discovery, Ministry of Education, and School of Pharmaceutical Sciences, Wuhan University, Wuhan, China; 3 Department of Respiratory and Critical Care Medicine, Zhongnan Hospital of Wuhan University, Key Laboratory of Combinatorial Biosynthesis and Drug Discovery, Ministry of Education and School of Pharmaceutical Sciences, Wuhan University, Wuhan, China; University of Virginia, UNITED STATES

## Abstract

Synthetic lethality (SL) occurs when mutations in two genes together lead to cell or organism death, while a single mutation in either gene does not have a significant impact. This concept can also be extended to three or more genes for SL. Computational and experimental methods have been developed to predict and verify SL gene pairs, especially for *yeast* and *Escherichia coli*. However, there is currently a lack of a specialized platform to collect microbial SL gene pairs. Therefore, we designed a synthetic interaction database for microbial genetics that collects 13,313 SL and 2,994 Synthetic Rescue (SR) gene pairs that are reported in the literature, as well as 86,981 putative SL pairs got through homologous transfer method in 281 bacterial genomes. Our database website provides multiple functions such as search, browse, visualization, and Blast. Based on the SL interaction data in the *S*. *cerevisiae*, we review the issue of duplications’ essentiality and observed that the duplicated genes and singletons have a similar ratio of being essential when we consider both individual and SL. The Microbial Synthetic Lethal and Rescue Database (Mslar) is expected to be a useful reference resource for researchers interested in the SL and SR genes of microorganisms. Mslar is open freely to everyone and available on the web at http://guolab.whu.edu.cn/Mslar/.

## Author summary

Research on SL interaction could provide novel drug targets and help construct a minimal genome. There have been many studies using metabolic models of different microbial systems or experimental array screen to study SL interactions. Here we developed a database to collect SL gene pairs of microorganisms, while also including SR gene pairs. Studies have transferred SL gene pairs onto other species through lineal homology, and also found that there are SL interactions. Based on this principle, we also used *Escherichia coli* as a reference set to calculate potential SL interactions among more than 2,700 bacteria, among which 281 have homologous SL gene pairs verified in *E*. *coli*.

## Introduction

Synthetic lethality (SR) is a genetic phenomenon where the combination of two mutations leads to cell death or inability to survive, while each mutation on its own does not have a significant impact [1]. Synthetic rescue (SR), on the contrary, describes a phenomenon that the expression defect of one gene will cause death, while the expression defect of two or more genes is non-lethal [2]. SL was first described by Calvin Bridges in 1922, who noted that certain combinations of mutations in the model organism Drosophila melanogaster were lethal [3]. Theodore Dobzhansky coined the term "synthetic lethality" in 1946 to describe the same type of genetic interaction in populations of wild-type fruit flies [4].

SL interactions have been studied in prokaryotes, such as model organisms *Escherichia coli*, as well as eukaryotes, such as *yeast*, fruit flies, nematodes, and so on [5–9]. The study of SL can further help people to understand complex biological systems, and it can be applied to the exploration of metabolic pathways and unknown functional genes. Research on microbial synthetic lethality would provide new clues for the discovery of antibacterial drug targets and the construction of chassis cell models [10–13].

Screening and discovery of SL interactions relied on mutation screening [14], nowadays, high-throughput screening has been applied to the analysis of SL, and synthetic genetic array (SGA) plays an important role in the construction of genome-scale double mutants [15]. SL has been extensively studied in *Escherichia coli* because it is an extremely important model organism with a widely available single-gene deletion collection (Keio) [16]. Costas D Maranas identified the SL pairs and SL triples using the genome-scale metabolic model of *E*. coli model iAF1260 [17]. Eric D. Brown *et al*. studied the SL interaction of *Escherichia coli* growing under nutritional stress [18], using 82 nutrient stress genes and Keio to create 315,400 double deletion mutants. And a total of 1,881 SL gene interactions were identified. Later, Eric D. Brown *et al*. crossed 53 shape-perturbing genes that expressed outer membrane or plasma membrane proteins with Keio to construct 1.7 million double deletion mutants, such that 1,373 SL interactions were screened out [19]. In 2016, Michael Costanzo et al. used synthetic genetic array (SGA) analysis to construct a comprehensive genetic interaction network of *S*. *cerevisiae*, constructing more than 23 million double mutants, from which about 550,000 negative interactions and 350,000 positive interactions were identified [20]. Adilson E Motter *et al*. identified more than 2,000 SR pairs of double deletion mutants through an alternative network-based strategy to force cells to bypass the functions affected by the defective genes or compensate for the lost function to restore biological function [21]. In addition to the studies on yeast and *Escherichia coli* SL, there have also been corresponding studies on *Bacillus subtilis*, *Candida albicans*, *Streptococcus agalactiae*, and *other microorganisms* [22–26]. Based on the above research, we developed Mslar to collect SL and SR data of microorganisms. In addition to providing basic functions of the database, we also provided visualization and prediction of possible SL gene pairs by homologous alignment in microorganisms.

## Results

Our database website provides multiple functionalities, including ‘Search’, ‘Browse’, ‘Visualization’, and ‘Blast’ options. All of the SL and SR data in the database can be downloaded directly. The statistics page displays summary information in detail as well as the reference literature. The help page introduces the functionalities of our database.

### Search

**Fig 1:**
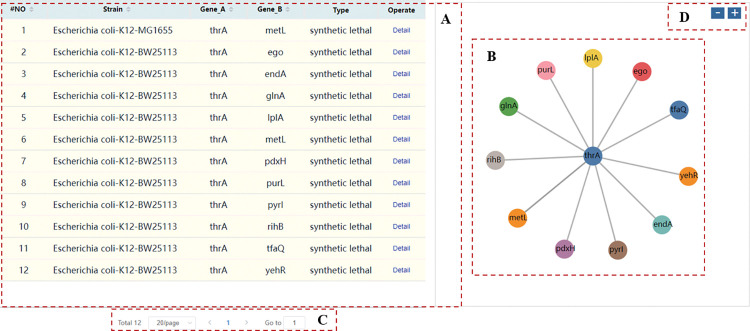
Search result for “thrA” gene. (A) A table displaying the results of the query gene. (B) The SVG shows the synthetic interaction with the query gene. (C) Pagination bar. (**D**) Buttons to zoom in or out the SVG.

Enter the gene name in the search box on the home page of the database website to search whether the gene has interaction data in our database. By default, the program will query for SL interactions from all the data we have collected. You can select the strain and interaction type to search. If the result returns, a table will be displayed on the left (Fig 1A), and a scalable vector graphics (SVG) visualization of gene interactions will be displayed on the right (Fig 1B), which allows users to visually observe the genes that interact with the resultant gene. The total number of results is displayed in the pagination bar (Fig 1C). Double-clicking any gene node in SVG will display the interaction data of the gene node, and the SVG will be updated at the same time. Click the button to zoom in or out of the SVG (Fig 1D). Fuzzy search is also supported. The help page has provided more information about the use of search.

### Browse

**Fig 2:**
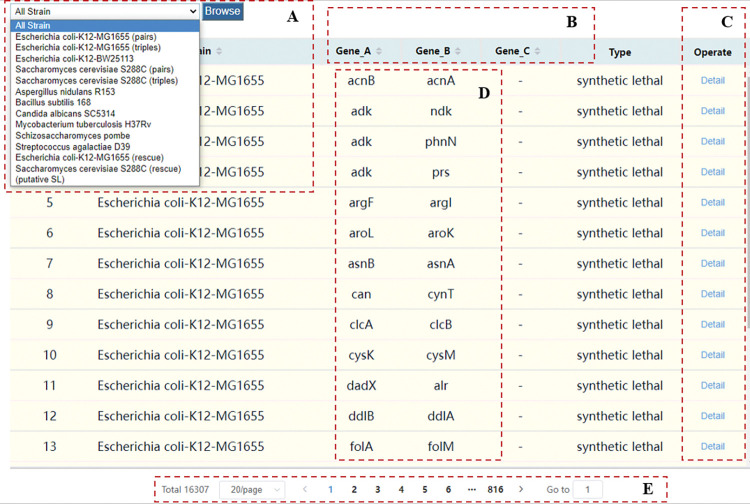
The default content of the browse page. (A) A drop-down box for selecting the data table. (B) Sort buttons. (C) Click the Detail button to view the details of this data. (D) Click on any gene name to display the interaction table and visualization of the gene. (E) Pagination bar.

**Fig 3:**
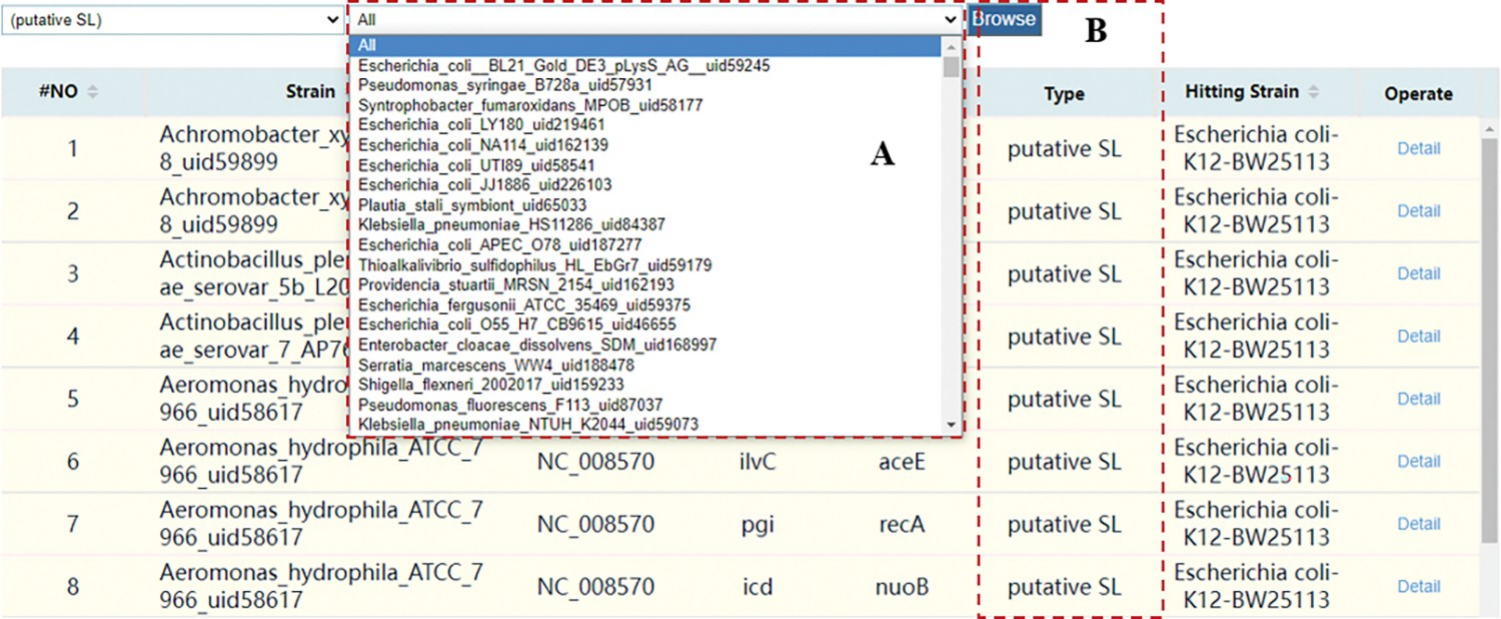
The putative SL of the browse page. (A) A drop-down box for selecting the Strain. (B) Click the ‘putative SL’ to view the homologous SL of this data.

By default, the Browse page displays SL and SR data collected from literature by us (Fig 2). You can select strain to browse data in the drop-down box (Fig 2A); selecting putative SL at the bottom of the drop-down box will load the putative SL interaction data we obtained by using Reciprocal Best Hit (RBH) alignment (Fig 3), and the page will load another drop-down box for strain selection browsing (Fig 3A). The RBH means aligning between two genomes and finding the best matching genes between them. In the data table, click the sort button on the title bar to sort the items and display them (Fig 2B). Click the Detail button to view the detailed information of this one interaction (Fig 2C), and click the gene name on the detail page to view the annotation information of the gene. Click any gene name to display the interaction table and visualization of the gene (Fig 2D), and the result is similar to Fig 1. Using the navigation bar at the bottom of the page you can select the number of pages and jump pages (Fig 2E). Click the ‘putative SL’ to view the homology hitting information of this putative SL interaction (Fig 3B).

Our database contains 13,313 SL interactions and 2,994 SR interactions. The interaction gene pairs of *S*. *cerevisiae* and *E*. *coli* account for a large proportion, which is because *E*. *coli* and the *yeast*, as the most critical model organisms, have been extensively studied by various researchers, including the study of SL effects. Among the 8,831 gene pairs of SL interactions in yeast collected by us, 2,997 genes were involved. And the highest number of genes having pairwise interactions with the same gene is 132, that is to say, this gene had SL interactions with a total of 132 genes. The 86,981 putative SL of 281 strains obtained by the homologous transfer method can also be viewed on the browse page.

### Blast

**Fig 4:**
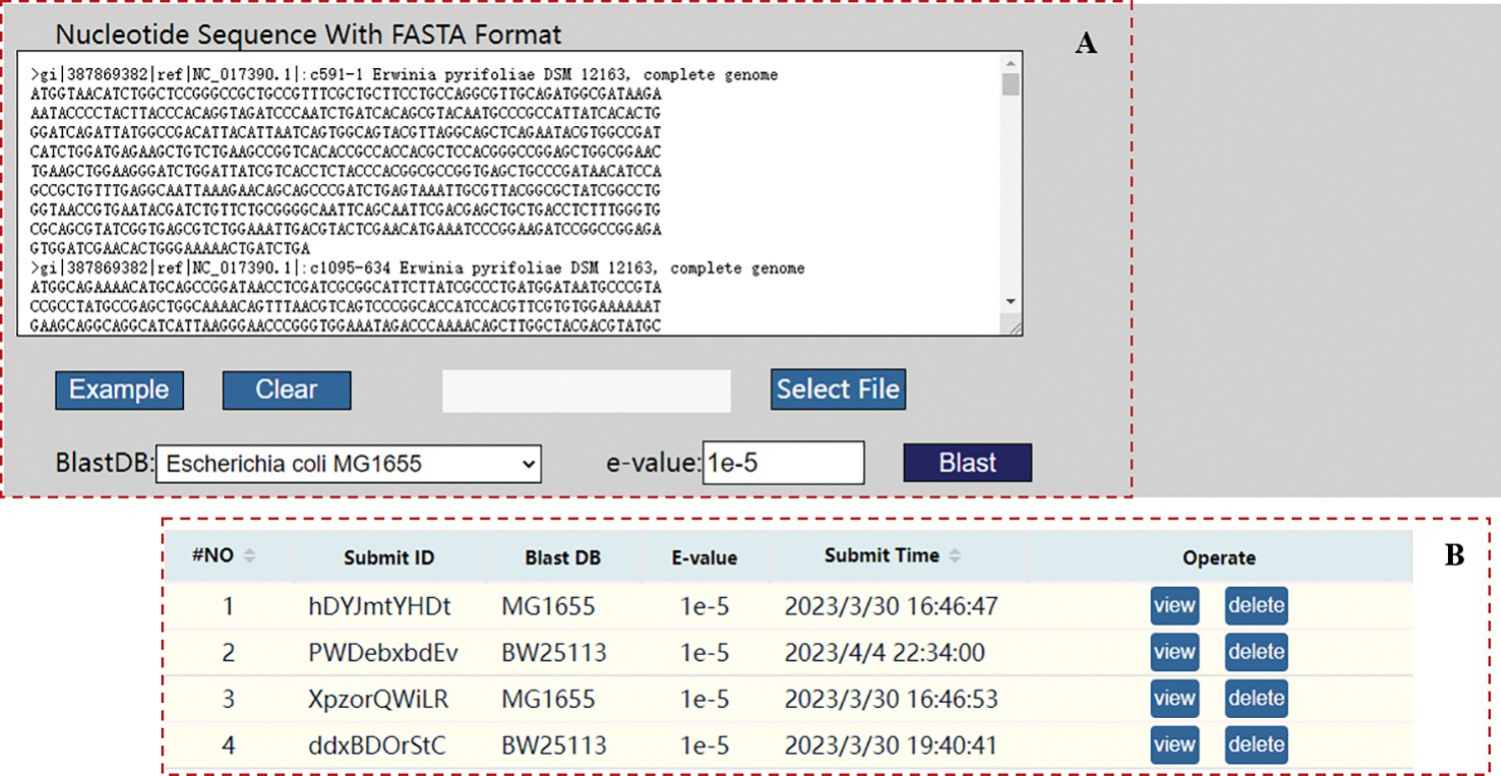
The Blast page. (**A**) The blast Box. (**B**) The records of submission.

Submit at least two FASTA nucleotide sequences on the Blast page (Fig 4A). Then, select the Blast reference library for alignment, click run, and the results will be displayed. If there is an error in the program, the page will prompt; otherwise, it will jump to the result page after the program finishes running. On the results page, the first data table displays successfully aligned genes and their corresponding alignment information, and the second data table displays potential SL gene pairs obtained by matching. Successful submissions will be shown in the record table (Fig 4B), and you can operate buttons to view results or delete a record.

## Discussion

There are already SL related databases, but these mostly focus on cancer synthetic lethality genes. SLKG (https://www.slkg.net/) provides an integrated platform that queries drug repositioning for tumor-specific therapy based on the concepts of synthetic lethality (SL) and synthetic dosage lethality (SDL). SynLethDB (https://synlethdb.sist.shanghaitech.edu.cn/) is a synthetic lethality database that aims to help cancer research by discovering selective and sensitive anti-cancer drug targets.

Our database focuses on synthetic lethal genes in microorganisms, and it also includes synthetic rescue genes. Compared to other comprehensive databases, ours is more specialized in the field of microbiology, allowing for a more detailed and in-depth exploration of synthetic lethality genes in microorganisms. Our database is oriented towards synthetic biology research, targeting combined drug targets of streamlined genomes of microbes. Among the unpublished putative SL pairs, we think there would be many corresponding to genuine SL, and they would help the functional genomic of the affiliated microbe after validating with the experimental method. The significance of these putative predictions could demonstrate only be demonstrated once they are validated in the future by related researchers. If there are indeed insightful applications in the future, we would like to share them as a case study when updating our database. We want to warn that these putative SL only serve as a reference for researchers, and if used in their work, experimental validation is highly recommended. In fact, when producing putative SL pair, we ask both genes have relatively higher similarity with the original literature-reported SL pair. That is to say, the e-value are both less than 1e-5 and identity larger than 25%. However, there may still exist false positive predictions among them. As details, now we provide alignment e-values and identity values between genes of resultant SL and original SL, and we hope this information could assist with judging the genuine SL for the users. Homology transfer has been applied in literature, and its basis is the observation that there are common SL between the S. cerevisiae and human [27]. If the two species have a closer evolutionary distance, the homology transfer of genetic interaction is more reliable. We listed the taxonomy relationship of the query species and the hit species for each putative SL.

Based on the complete list of synthetic gene pairs in *S*. *cerevisiae*, we performed an analysis of the typical characteristics of these interactions. First, it involved a distance between genes with pairwise interaction and limited the analysis to those genes located on the same chromosome. For each chromosome, we calculated the average distance between all paired genes. With 16 chromosomes, we made a linear regression between the interaction distance and chromosome length, and a strong correlation was shown (R = 0.92, p = 6.38–07).

Secondly, duplicated genes have been observed to be less essential than single genes according to the ratios of being essential in these two types of genes. In 2003, Gu and his colleagues observed that the proportion of essential genes (PE) among duplicates is much lower than among singletons in yeast [28]. However, subsequently, contradicting results in mice were encountered [29]. Liao and Zhang found that the proportion of essentials among duplicates is comparable to that among singletons. Two follow-up studies [30,31] discovered that the knockout data were further enriched in genes derived from old duplications and in developmental genes; after correcting these biases, the overall PE in duplicates became statistically significantly lower than that in singletons. In 2012, Chen and colleagues confirmed the above result and found that at a given phyletic gene age, duplicates are always less likely to be essential compared with singletons [32]. So far, the reason duplicates appear to be less essential than singletons has not been made clear. Here, we try to clarify this issue based on gene essentiality and genetic interaction in *S*. *cerevisiae*. There are a total of 1110 essential genes and 9225 synthetic lethal pairs in *S*. *cerevisiae*. On the other hand, this species has 1152 duplicates [33,34] and the rest 5564 genes are singletons. If we limit the essentiality only to single genes’ effect, the duplications will have a much less essentiality ratio than that of singletons (76/1152 = 0.066 vs 1034/5564 = 0.1858), where the essentiality ratio is the number of genes being essential for duplicates and singletons divided by the number of either type of the genes. However, synthetic gene pairs also have lethal effects and if we also consider such essentiality, the result would be the opposite. For singletons, they have 46.92% chance to be involved in synthetical lethal and the probability of being individually essential and synthetically essential would be 66.6%. Interestingly, the same probability (0.066+0.60 = 0.666) is obtained for duplications. Therefore, when we consider both the individually essential and SL, the probability of essential genes (gene pair) would be similar between singleton and duplicated genes.

## Methods

### Data source

By searching the keyword "synthetic lethality" in the NCBI PubMed database, we obtained hundreds of publications about SL. After reading abstracts, we screened out literature related to microbial SL gene, and then carefully read more than 20 pieces of literature to find the data we need. The data were obtained by the text mining method. We then screened out the data we were interested in according to the methods and reference indexes provided by the literature. Most of the SL genes in our database belong to Saccharomyces cerevisiae and Escherichia coli, which is also because these two are the most critical model microbes of eukaryotic and prokaryotic. After obtaining the SL data, we downloaded and processed the gene annotation information of corresponding microorganisms from genome data websites (GenBank and SGD). And added this data to our database to provide more complete data information. In the SL data items collected by us, original important information and PMID are saved, so that users can view the detailed information of SL gene pairs and explore the research methods of SL in literature. The data we collected are shown in Table 1.

**Table 1:** SL and SR data from the literature.

Genome	Type	Pair Number	Gene Number
*Escherichia coli* K12-MG1655	SL	70	85
*Escherichia coli* K12-MG1655	SL(triples)	157	109
*Escherichia coli* K12-MG1655	SR	2423	180
*Escherichia coli* K12-BW25113	SL	3254	890
*Saccharomyces cerevisiae*	SL	8831	2997
*Saccharomyces cerevisiae*	SL(triples)	991	691
*Saccharomyces cerevisiae*	SR	562	99
*Aspergillus nidulans* R153	SL	1	2
*Bacillus subtilis* 168	SL	2	4
*Candida albicans* SC5314	SL	3	5
*Mycobacterium tuberculosis* H37Rv	SL	1	2
*Schizosaccharomyces pombe*	SL	2	4
*Streptococcus agalactiae* D39	SL	1	2

This is a table listing the SL and SR data we collected and curated. The ‘triples’ stands for SL of three genes. The column of ‘Pair Number’ shows the number of SL interactions. The column ‘Gene Number’ shows the number of genes involved with SL interaction in that genome. Note that we also produced 86,981 putative SL gene pairs in 281 bacterial genomes by homologous transfer.

### Database and web

After consistently handling the original data, we used MySQL to build the database. And we designed the responsive website in combination with HTML, CSS, JavaScript, and Vue. The force-directed Graph of D3.js (https://d3js.org/) was applied to realize the visual node interactive Graph of gene interaction. We also used PHP for database access and Python for data processing. All major browsers support access to our database website.

### Putative synthetic lethality

Due to the genome integrity and the highly conserved nature of genes related to the cell cycle, previous studies have transferred orthologous genes of SL in microorganisms to other organisms through comparative genomics [27,35,36]. Here, we used two strains of *E*. *coli* and *Saccharomyces cerevisiae* to build the reference library. And then used RBH [37] alignment for 2,700 prokaryotic genomes to get orthologous SL pairs. For these putative SL pairs, we supplement our original SL data in our database with them.

## Supporting information

S1 Text An introduction to how to use the functionalities of the Microbial Synthetic Lethal and Rescue Database. (PDF)
