# Supplementary material for: Mslar: Microbial synthetic lethal and rescue database
Source: PLoS Comput Biol. 2023 Jun 8;19(6):e1011218. doi: 10.1371/journal.pcbi.1011218 (PMC10284384; doi:10.1371/journal.pcbi.1011218)
Supplement: S1 Text — (PDF) [file pcbi.1011218.s001.pdf]

Functionalities of Microbial Synthetic Lethal and Rescue Database

1. Search

Enter the gene name in the search box (**Figure S1**) on the Home page of the database website (http://guolab.whu.edu.cn/Mslar/) to search whether one gene has interaction data in our database. By default, The program will search for synthetic lethal (SL) from the collected data. You can select the strain and interaction type to search. Letter-case is ignored when searching. The characters you can enter including [A-Za-z0-9%_-]. If any other characters are entered, an error will be reported.

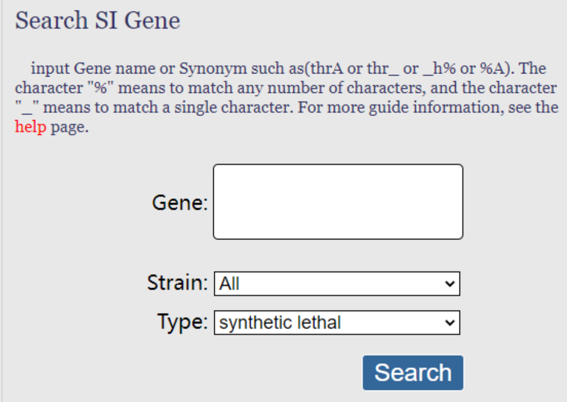

Figure S1. The Search Box.

Enter the '**thrA**' in the search box, the search results are shown in **Figure S2**. A table of results is shown on the left, and a visual scalable vector graphics (SVG) of the interaction between genes is shown on the right. Click the sort button to realize the sort (**Figure S2 A**). Click the gene name in the result table to view the search result of the gene be clicked (**Figure S2 B**). In the pagination bar, you can view the total number of results and jump to the page which you want to view (**Figure S2 C**). Click the Detail button to view more information on the data (**Figure S2 D**), the result will be shown in **Figure S3**. Double-click the gene node in SVG to view the search results of the gene be clicked. In addition, you can drag a gene node with the mouse (**Figure S2 E**). Click the - or + button to zoom in or out of the SVG (**Figure S2 F**). Fuzzy search is also supported. More guidance on search can be found on the help page on our website.

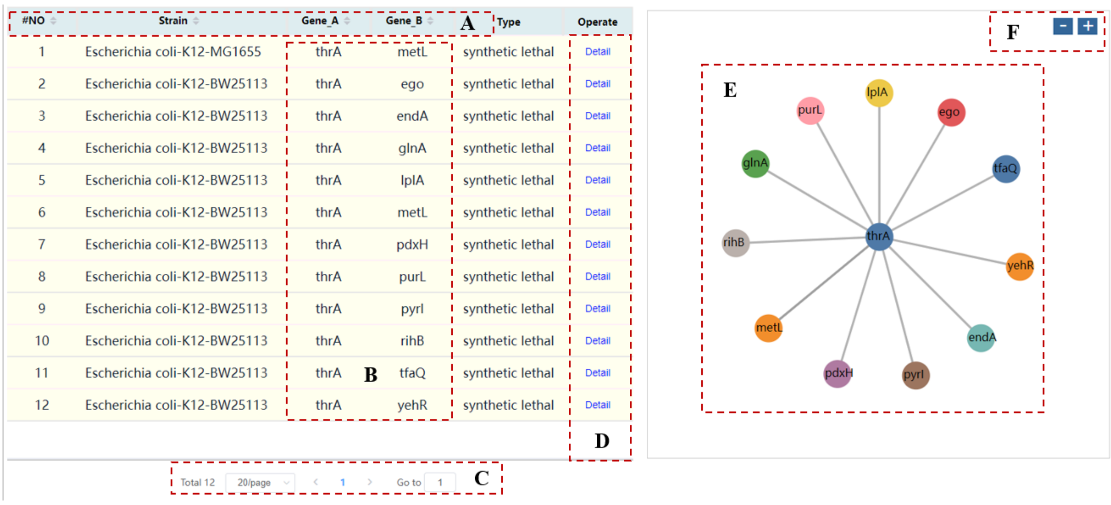

#NO	Strain	Gene_A	Gene_B	Type	Operate
1	Escherichia coli-K12-MG1655	thrA	metL	synthetic lethal	Detail
2	Escherichia coli-K12-BW25113	thrA	ego	synthetic lethal	Detail
3	Escherichia coli-K12-BW25113	thrA	endA	synthetic lethal	Detail
4	Escherichia coli-K12-BW25113	thrA	glnA	synthetic lethal	Detail
5	Escherichia coli-K12-BW25113	thrA	lplA	synthetic lethal	Detail
6	Escherichia coli-K12-BW25113	thrA	metL	synthetic lethal	Detail
7	Escherichia coli-K12-BW25113	thrA	pdxH	synthetic lethal	Detail
8	Escherichia coli-K12-BW25113	thrA	purL	synthetic lethal	Detail
9	Escherichia coli-K12-BW25113	thrA	pyrI	synthetic lethal	Detail
10	Escherichia coli-K12-BW25113	thrA	rihB	synthetic lethal	Detail
11	Escherichia coli-K12-BW25113	thrA	tfaQ	synthetic lethal	Detail
12	Escherichia coli-K12-BW25113	thrA	yehR	synthetic lethal	Detail

Figure S2. The search results for the "thrA" gene.

Strain	Escherichia coli-K12-MG1655
Gene_A	thrA
Gene_A Synonym	ECK0002|Hs|JW0001|thrA1|thrA2|thrD
Gene_B	metL
Gene_B Synonym	ECK3932|JW3911|metM
PMID	19690570
Synthetic lethal method	in silico multiple knockout
Experiment condition	in silico work based on the iAF1260 model of E. coli K12
Type	synthetic lethal

Figure S3. SL for "thrA" and "metL".

Click the gene name in **Figure S3** to view the gene annotation information. The result is shown in **Figure S4.** Click PMID in **Figure S3** to view the reference literature.

genomelink	U00096.3
gene	thrA
locus_tag	b0002
gene_synonym	ECK0002|Hs|JW0001|thrA1|thrA2|thrD
EcoGene	EG10998
location	337..2799
gbkey	Gene
product	Bifunctional aspartokinase/homoserine dehydrogenase 1
protein_id	AAC73113.1
translation	MRVLKFGGTSVANAERFLRVADILESNARQGQVATVLSAPAKITNHLVAMIEKTISGQDALPNISDAERIFAELLTGLAAAQPGFPLAQLKTFVDQEFAQIKHVLHGISLLGQCPDSIN<br>AALICRGEKMSIAIMAGVLEARGHNVTVIDPVEKLLAVGHYLESTVDIAESTRRIAASRIPADHMVLMAGFTAGNEKGELVVLGRNGSDYSAAVLAACLRADCCEIWTDVDGVYTCD<br>PRQVPDARLLKSMSYQEAMELSYFGAKVLHPRTITPIAQFQIPCLIKNTGNPQAPGTLIGASRDEDELPVKGISNLNNMAMFSVSGPGMKGMVGMAARVFAAMSRARISVVLITQS<br>SSEYSISFCVPQSDCVRAERAMQEEFYLELKEGLLEPLAVTERLAIISVVGDGMRTLRGISAKFFAALARANINIVAIAQGSSERSISVVVNNDDATTGVRVTHQMLFNTDQVIEVFVIG<br>VGGVGGALLEQLKRQQSWLKNKHIDLRVCGVANSKALLTNVHGLNLENWQEELAQAKEPFNLGRLIRLVKEYHLLNPVIVDCTSSQAVADQYADFLREGFHVVTPNKKANTSSM<br>DYYHQLRYAAEKSRRKFLYDTNVGAGLPVIENLQNLLNAGDELMKFSGILSGSLSYIFGKLDEGMSFSEATTLAREMGYTEPDPRDDLSGMDVARKLLILARETGRELELADIEIEPVLP<br>AEFNAEGDVAAFMANLSQLDDLFAARVAKARDEGKVLRYVGNIDEDGVCRVKIAEVDGNDPLFKVKNGENALAFYSHYYQPLPLVLRGYGAGNDVTAAGVFADLLRTLSWKLGV
Gene_sequence	ATGCGAGTGTTGAAGTTCGGCGGTACATCAGTGGCAAATGCAGAACGTTTTCTGCGTGTTGCCGATATTCTGGAAAGCAATGCCAGGCAGGGGCAGGTGGCCACCGTCCTCTCT<br>GCCCCCGCCAAAATCACCAACCACCTGGTGGCGATGATTGAAAAAACCATTAGCGGCCAGGATGCTTTACCCAATATCAGCGATGCCGAACGTATTTTTGCCGAACTTTTGACG<br>GGACTCGCCGCCGCCCAGCCGGGGTTCCCGCTGGCGCAATTGAAAACTTTCGTCGATCAGGAATTTGCCCAAATAAAACATGTCCTGCATGGCATTAGTTTGTTGGGGCAGTGC<br>CCGGATAGCATCAACGCTGCGCTGATTTGCCGTGGCGAGAAAATGTCGATCGCCATTATGGCCGGCGTATTAGAAGCGCGCGGTCACAACGTTACTGTTATCGATCCGGTCGAA<br>AAACTGCTGGCAGTGGGGCATTACCTCGAATCTACCGTCGATATTGCTGAGTCCACCCGCCGTATTGCGGCAAGCCGCATTCCGGCTGATCACATGGTGCTGATGGCAGGTTTCA<br>CCGCCGGTAATGAAAAAGGCGAACTGGTGGTGCTTGGACGCAACGGTTCCGACTACTCTGCTGCGGTGCTGGCTGCCTGTTTACGCGCCGATTGTTGCGAGATTTGGACGGAC<br>GTTGACGGGGTCTATACCTGCGACCCGCGTCAGGTGCCCGATGCGAGGTTGTTGAAGTCGATGTCCTACCAGGAAGCGATGGAGCTTTCCTACTTCGGCGCTAAAGTTCTTCAC<br>CCCCGCACCATTACCCCCATCGCCCAGTTCCAGATCCCTTGCCTGATTAAAAATACCGGAAATCCTCAAGCACCAGGTACGCTCATTGGTGCCAGCCGTGATGAAGACGAATTAC<br>CGGTCAAGGGCATTTCCAATCTGAATAACATGGCAATGTTCAGCGTTTCTGGTCCGGGGATGAAAGGGATGGTCGGCATGGCGGCGCGCGTCTTTGCAGCGATGTCACGCGCC<br>CGTATTTCCGTGGTGCTGATTACGCAATCATCTTCCGAATACAGCATCAGTTTCTGCGTTCCACAAAGCGACTGTGTGCGAGCTGAACGGGCAATGCAGGAAGAGTTCTACCTGG<br>AACTGAAAGAAGGCTTACTGGAGCCGCTGGCAGTGACGGAACGGCTGGCCATTATCTCGGTGGTAGGTGATGGTATGCGCACCTTGCGTGGGATCTCGGCGAAATTCTTTGCC<br>GCACTGGCCCGCGCCAATATCAACATTGTCGCCATTGCTCAGGGATCTTCTGAACGCTCAATCTCTGTCGTGGTAAATAACGATGATGCGACCACTGGCGTGCGCGTTACTCATC<br>AGATGCTGTTCAATACCGATCAGGTTATCGAAGTGTTTGTGATTGGCGTCGGTGGCGTTGGCGGTGCGCTGCTGGAGCAACTGAAGCGTCAGCAAAGCTGGCTGAAGAATAAA<br>CATATCGACTTACGTGTCTGCGGTGTTGCCAACTCGAAGGCTCTGCTCACCAATGTACATGGCCTTAATCTGGAAAACTGGCAGGAAGAACTGGCGCAAGCCAAAGAGCCGTTT<br>AATCTCGGGCGCTTAATTCGCCTCGTGAAAGAATATCATCTGCTGAACCCGGTCATTGTTGACTGCACTTCCAGCCAGGCAGTGGCGGATCAATATGCCGACTTCCTGCGCGAAG<br>GTTTCCACGTTGTCACGCCGAACAAAAAGGCCAACACCTCGTCGATGGATTACTACCATCAGTTGCGTTATGCGGCGGAAAAATCGCGGCGTAAATTCCTCTATGACACCAACG<br>TTGGGGCTGGATTACCGGTTATTGAGAACCTGCAAAATCTGCTCAATGCAGGTGATGAATTGATGAAGTTCTCCGGCATTCTTTCTGGTTCGCTTTCTTATATCTTCGGCAAGTTA<br>GACGAAGGCATGAGTTTCTCCGAGGCGACCACGCTGGCGCGGGAAATGGGTTATACCGAACCGGACCCGCGAGATGATCTTTCTGGTATGGATGTGGCGCGTAAACTATTGAT<br>TCTCGCTCGTGAAACGGGACGTGAACTGGAGCTGGCGGATATTGAAATTGAACCTGTGCTGCCCGCAGAGTTTAACGCCGAGGGTGATGTTGCCGCTTTTATGGCGAATCTGTC<br>ACAACTCGACGATCTCTTTGCCGCGCGCGTGGCGAAGGCCCGTGATGAAGGAAAAGTTTTGCGCTATGTTGGCAATATTGATGAAGATGGCGTCTGCCGCGTGAAGATTGCCG<br>AAGTGGATGGTAATGATCCGCTGTTCAAAGTGAAAAATGGCGAAAACGCCCTGGCCTTCTATAGCCACTATTATCAGCCGCTGCCGTTGGTACTGCGCGGATATGGTGCGGGCA<br>ATGACGTTACAGCTGCCGGTGTCTTTGCTGATCTGCTACGTACCCTCTCATGGAAGTTAGGAGTCTGA

Figure S4. Gene annotation information

2. Browse

By default, the Browse page displays SL and SR data collected from literature by us (**Figure S5**). You can select different types of data in the drop-down box to browse synthetic lethal or rescue data of a strain (**Figure S5 A**). In the data table, click the sort button in the title bar to sort the items and display them (**Figure S5 B**). Click the Detail button to view the detailed information on this one interaction (**Figure S5 C**), and the result is similar to that in **Figure S3**. Click any gene name to display the interaction table and visualization of the gene (**Figure S3 D**), and the result is shown similarly in **Figure S2**. You can select the size of the page and jump page in the bottom pagination bar (**Figure S5 E**).

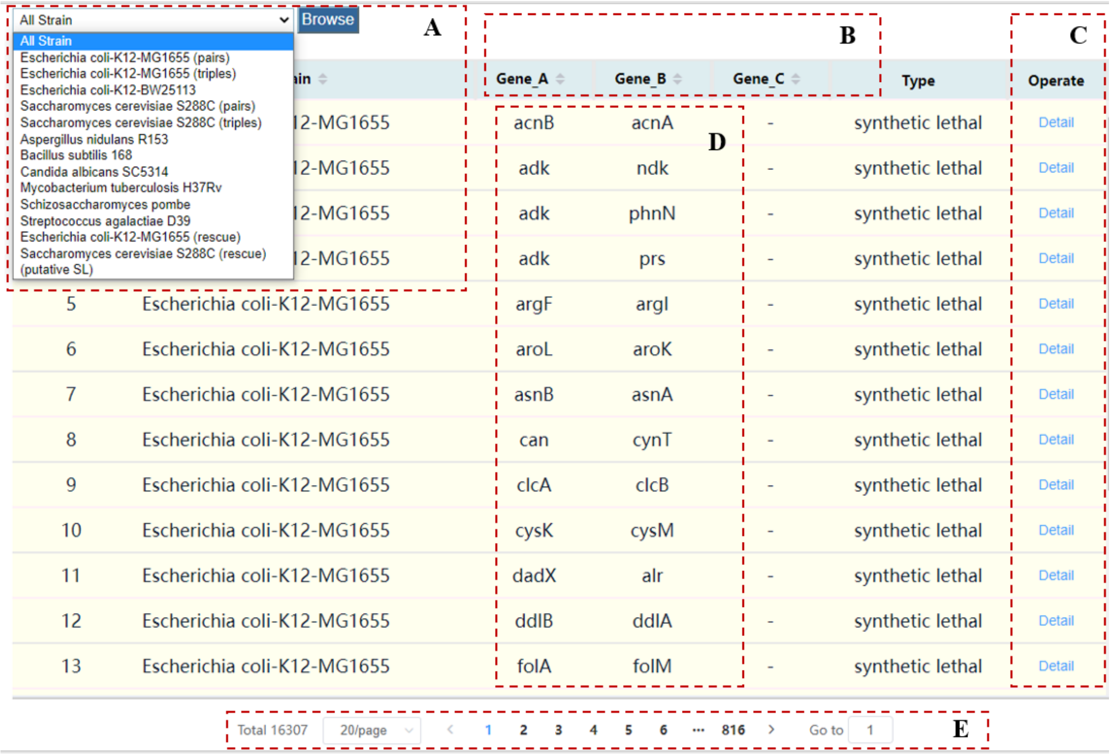

Figure S5. The default content of the Browse page.

Selecting the option ‘putative SL’ at the bottom of the drop-down box **(Figure S5 A)** will load the putative SL data **(Figure S6)**, and the page will load another drop-down box **(Figure S6 A)** for strain selection browsing. Click the "putative SL" in **(Figure S6 B)** which will show the homologous synthetic lethal pairs.

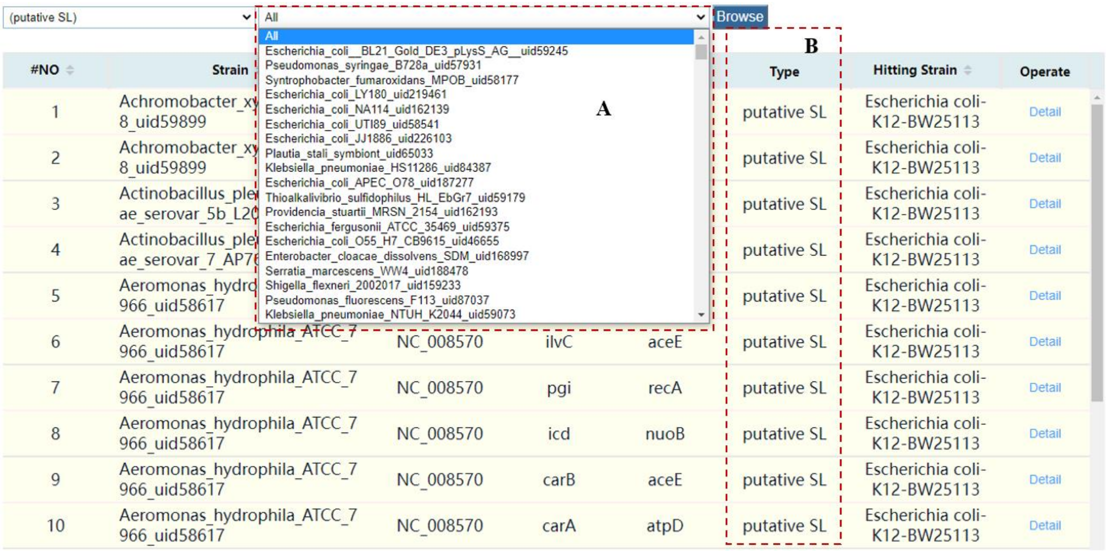

Figure S6.Putative SL data.

3. Blast

Submit at least two FASTA nucleotide sequences on Blast Box (**Figure S7 A**). Click the 'Example' button to view the example of the FASTA format. Click the 'Clear' button to clear the text area box. Click the 'Select File' button to upload a local file in FASTA format. Select the blast reference library for alignment, then click the 'Blast' button to run, and the results will be displayed. If there is an error in the program, the page will prompt, otherwise, it will jump to the result page after the program finishes running. The successful submission will be displayed in the table (**Figure S7 B**). Click the 'view' button to view the result of the record. Click the 'delete' button to delete one record. For example, we run the example with the '*Escherichia coli* BW25113' in BlastDB, on the result page, the first data table displays successfully aligned genes and their corresponding alignment information (**Figure S8**), and the second data table displays potential synthetic lethal gene pairs obtained by homologous transfer (**Figure S9**).

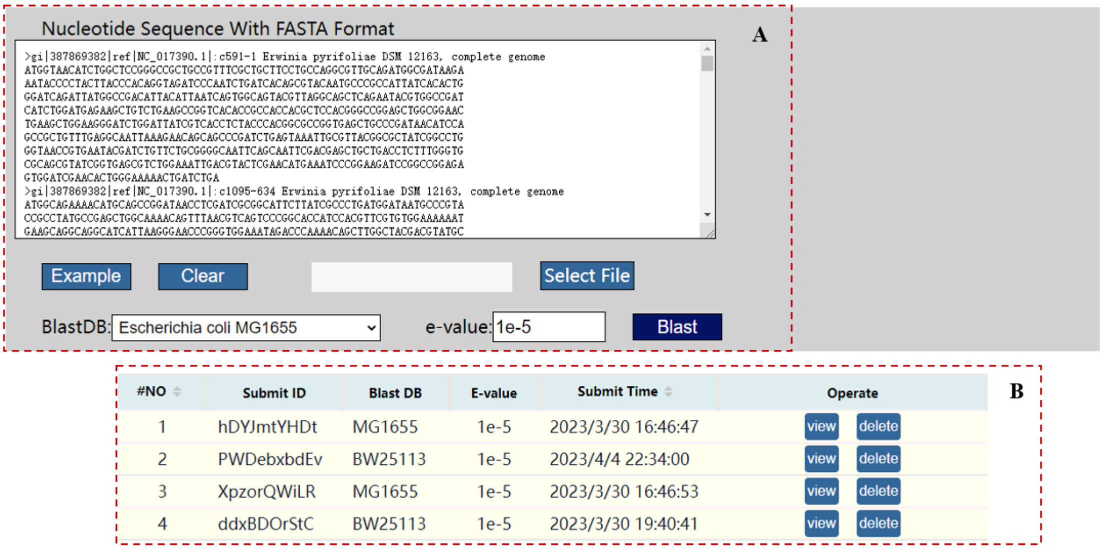

Figure S7. Blast page.

Blast Result

#NO	Query_ID	Hit_ID	Identity	align length	mismatches	gaps	query_start	query_end	hit_
1	gi|387869382|ref|NC_017390.1|:c50793-49744	CP009273.1_cds_AIN34172.1_3794	77.874	1044	223	7	1	1040	
2	gi|387869382|ref|NC_017390.1|:c52393-50984	CP009273.1_cds_AIN34173.1_3795	83.463	1415	224	10	1	1410	
3	gi|387869382|ref|NC_017390.1|:52829-54652	CP009273.1_cds_AIN34174.1_3796	84.031	1791	278	8	1	1787	
4	gi|387869382|ref|NC_017390.1|:181107-182030	CP009273.1_cds_AIN34259.1_3882	82.238	867	146	8	1	863	
5	gi|387869382|ref|NC_017390.1|:199203-201053	CP009273.1_cds_AIN34088.1_3709	79.794	1841	356	13	1	1833	
6	gi|387869382|ref|NC_017390.1|:203653-205131	CP009273.1_cds_AIN34091.1_3712	82.963	1485	238	11	1	1479	
7	gi|387869382|ref|NC_017390.1|:205488-207581	CP009273.1_cds_AIN34093.1_3715	79.738	1984	374	24	73	2042	
8	gi|387869382|ref|NC_0173[illegible]	CP009273.1_cds_AIN3409[illegible]	85.726	1261	178	2	1	1260	

Total 113 20/page < 1 2 3 4 5 6 > Go to 1

Figure S8. An example of the blast results.

#NO	Query_A	Hit_ID_A	Hit_Gene_A	Query_B	Hit_ID_B	Hit_Gene_B	Operate
1	gi|387869382|ref|NC_017390.1|:c50793-49744	CP009273.1_cds_AIN34172.1_3794	glnL	gi|387869382|ref|NC_017390.1|:c52393-50984	CP009273.1_cds_AIN34173.1_3795	glnA	Detail
2	gi|387869382|ref|NC_017390.1|:c52393-50984	CP009273.1_cds_AIN34173.1_3795	glnA	gi|387869382|ref|NC_017390.1|:565729-567378	CP009273.1_cds_AIN34430.1_4056	groL	Detail
3	gi|387869382|ref|NC_017390.1|:c52393-50984	CP009273.1_cds_AIN34173.1_3795	glnA	gi|387869382|ref|NC_017390.1|:812636-815452	CP009273.1_cds_AIN30563.1_25	ileS	Detail
4	gi|387869382|ref|NC_017390.1|:c52393-50984	CP009273.1_cds_AIN34173.1_3795	glnA	gi|387869382|ref|NC_017390.1|:c848622-847225	CP009273.1_cds_AIN30605.1_69	leuC	Detail
5	gi|387869382|ref|NC_017390.1|:c52393-50984	CP009273.1_cds_AIN34173.1_3795	glnA	gi|387869382|ref|NC_017390.1|:c851330-849717	CP009273.1_cds_AIN30607.1_71	leuA	Detail
6	gi|387869382|ref|NC_017390.1|:c52393-50984	CP009273.1_cds_AIN34173.1_3795	glnA	gi|387869382|ref|NC_017390.1|:918895-920493	CP009273.1_cds_AIN30645.1_109	aceF	Detail
7	gi|387869382|ref|NC_017390.1|:c52393-50984	CP009273.1_cds_AIN34173.1_3795	glnA	gi|387869382|ref|NC_017390.1|:1143499-1144155	CP009273.1_cds_AIN32975.1_2552	rseA	Detail
8	gi|387869382|ref|NC_017390.1|:c52393-50984	CP009273.1_cds_AIN34173.1_3795	glnA	gi|387869382|ref|NC_017390.1|:2598458-2599117	CP009273.1_cds_AIN31279.1_790	glnP	Detail
9	gi|387869382|ref|NC_017390.1|:c52393-50984	CP009273.1_cds_AIN34173.1_3795	glnA	gi|387869382|ref|NC_017390.1|:2599114-2599836	CP009273.1_cds_AIN31278.1_789	glnQ	Detail
10	gi|387869382|ref|NC_017390.1|:c52393-50984	CP009273.1_cds_AIN34173.1_3795	glnA	gi|387869382|ref|NC_017390.1|:c3068058-3066991	CP009273.1_cds_AIN33074.1_2658	recA	Detail
11	gi|387869382|ref|NC_017390.1|:c52393-50984	CP009273.1_cds_AIN34173.1_3795	glnA	gi|387869382|ref|NC_017390.1|:c3158009-3154641	CP009273.1_cds_AIN33194.1_2778	recC	Detail
12	gi|387869382|ref|NC_01739[illegible]	CP009273.1_cds_AIN34173.1[illegible]	glnA	gi|387869382|ref|NC_01739[illegible]	CP009273.1_cds_AIN33307.1[illegible]	gshB	Detail

Figure S9. An example of the results of putative synthetic lethal gene pairs.
